# Chronic Wound Initiation: Single-Cell RNAseq of Cutaneous Wound Tissue and Contributions of Oxidative Stress to Initiation of Chronicity

**DOI:** 10.3390/antiox14020214

**Published:** 2025-02-13

**Authors:** Parnian Jabbari, Jane H. Kim, Brandon H. Le, Wei Zhang, Huimin Zhang, Manuela Martins-Green

**Affiliations:** 1 Department of Molecular, Cell, and Systems Biology, University of California, Riverside, CA 92521, USA; pjabb001@ucr.edu (P.J.); jkim193@ucr.edu (J.H.K.); huimin.zhang@ucr.edu (H.Z.); 2 Institute of Integrative Genome Biology, University of California, Riverside, CA 92521, USA; lbrandon@ucr.edu (B.H.L.); wezhang@ucr.edu (W.Z.); 3 Department of Botany and Plant Sciences, University of California, Riverside, CA 92521, USA

**Keywords:** healing, cellular heterogeneity, wound microenvironment, oxidative stress, diabetic foot ulcers, animal models

## Abstract

Chronic wounds (CWs) in humans affect millions of people in the US alone, cost billions of dollars, cause much suffering, and still there are no effective treatments. Patients seek medical care when wound chronicity is already established, making it impossible to investigate factors that initiate chronicity. In this study, we used a diabetic mouse model of CWs that mimics many aspects of chronicity in humans. We performed scRNAseq to compare the cell composition and function during the first 72 h post-injury and profiled 102,737 cells into clusters of all major cell types involved in healing. We found two types of fibroblasts. Fib 1 (pro-healing) was enriched in non-CWs (NCWs) whereas Fib 2 (non-healing) was in CWs. Both showed disrupted proliferation and migration, and extracellular matrix (ECM) deposition in CWs. We identified several subtypes of keratinocytes, all of which were more abundant in NCWs, except for Channel-related keratinocytes, and showed altered migration, apoptosis, and response to oxidative stress (OS) in CWs. Vascular and lymphatic endothelial cells were both less abundant in CWs and both had impaired migration affecting the development of endothelial and lymphatic microvessels. Study of immune cells showed that neutrophils and mast cells are less abundant in CWs and that NCWs contained more proinflammatory macrophages (M1) whereas CWs were enriched in anti-inflammatory macrophages (M2). Also, several genes involved in mitochondrial function were abnormally expressed in CWs, suggesting impaired mitochondrial function and/or higher OS. Heat shock proteins needed for response to OS were downregulated in CWs, potentially leading to higher cellular damage. In conclusion, the initiation of chronicity is multifactorial and involves various cell types and cellular functions, indicating that one type of treatment will not fix all problems, unless the root cause is fundamental to the cell and molecular mechanisms of healing. We propose that such a fundamental process is high OS and its association with wound infection/biofilm.

## 1. Introduction

**Figure 1:**
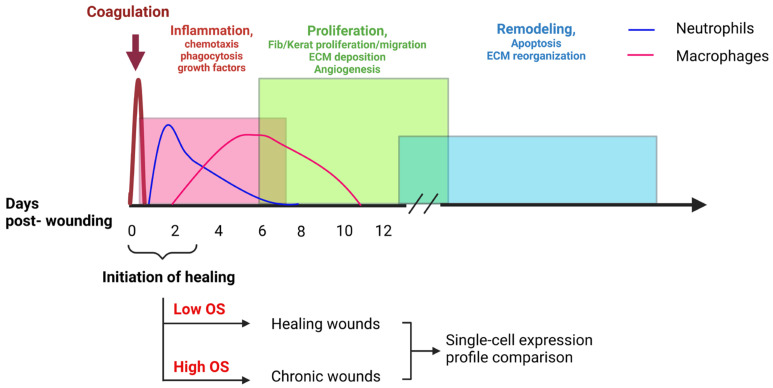
Normal process of wound healing. The wounds heal through the overlapping stages of coagulation, inflammation, proliferation, and remodeling. We focused on the very early times (the first 3 days) post-wound to determine the role of each cell type and their cellular and molecular processes in initiation of chronicity.

Skin is the largest organ of the human body that protects us from environmental hazards [1]. It has three layers: the outermost layer, epidermis, is primarily composed of keratinocytes, with the top layer being cornified and sloughed off, which will be replaced by new cells differentiating from basal keratinocytes. The middle layer, dermis, is mostly composed of fibroblasts, surveilling immune cells, microvessels, and an extracellular matrix (ECM) rich in collagen, which confers strength and flexibility to the skin. The innermost layer, hypodermis, connects the upper layers to the underlying connective tissue [2]. Once an injury occurs in the skin, oxidative stress (OS) increases and is followed by a series of orderly and timely overlapping processes that take place to seal the wound and restore the integrity of the skin. These processes can be categorized into four phases: hemostasis, inflammatory phase, proliferative phase, and remodeling phase [2]. During homeostasis, blood vessels constrict, and platelets aggregate to form a clot with a fibrin mesh that stops bleeding by temporarily sealing the wound and acting as a scaffold for the incoming cells. During the inflammatory phase, immune cells, in particular neutrophils and macrophages, are recruited to the injury site to kill pathogens and clean up the cellular debris and in the process increase OS in the wound tissue. The immune cells secrete cytokines that will signal fibroblasts, endothelial cells, and keratinocytes to proliferate and migrate to heal the wound. These processes are required to form the granulation tissue and new blood vessels to carry in oxygen and remove waste and to re-epithelialize the wound during the proliferative phase [3,4]. Even though wounds can be considered healed after re-epithelialization, the healing process continues for several months through the remodeling phase. In this phase, the balance between matrix metalloproteinases (MMPs) and tissue inhibitors of metalloproteinases (TIMPs) remodel the ECM and eventually generate scar tissue (Figure 1) [5]. Any deviation from these processes, either in time or order in which they take place or increase in OS or changes in the composition of cells or their functions in the wound microenvironment, can lead to delayed healing or wound chronicity [6].

Chronic wounds (CWs) are a global burden, with 10.5 million people affected in the United States alone [7]. These wounds can be divided into different categories based on their etiology. One category, diabetic foot ulcers (DFUs), is a major class of CWs affecting 30% of an estimated 537 million people with diabetes worldwide, and given the prevalence of sedentary lifestyles, these numbers are only expected to grow. Current treatments of DFUs include debridement of the ulcer along with appropriate antibiotic treatment, use of different growth factors or bioengineered scaffolds and skin grafts, and, in some cases, adjunct treatment, such as negative-pressure wound therapy, to promote healing of the wounds [8,9,10]. Despite these aggressive treatments, CWs fail to heal or heal temporarily and often reopen. What has not been studied is how OS contributes to the cellular and molecular changes that occur when wound chronicity ensues. This requires understanding the differences between healing wounds (NCWs) and CWs at very early stages after injury. This lack of understanding is in part due to the fact that patients do not seek medical care until chronicity of the wound is already established, making it hard to study the initial stages of healing or chronicity in humans, hence requiring reliable animal models to perform comparative studies between healing and non-healing (chronic) wounds.

*This study is novel* because we compare the role of different cells and of differential gene expression in the initiation of chronicity, not when the wounds are fully chronic, as has been performed before. Here, we compared the transcriptomic landscape of NWCs, which contain low levels of OS, with those of CWs, which contain high levels of OS, in our diabetic mice model of CWs at the very early stages of the response to injury (Figure 1) [11]. To replicate the high levels of OS resulting from high glucose levels and depleted glutathione reservoirs in diabetic patients [12], diabetic mice were treated with inhibitors of two of the main antioxidant enzymes, catalase and glutathione peroxidase [11]. This manipulation leads to the formation of CWs 100% of the time. We have shown previously that the high levels of OS also contribute to the natural development of a bacterial biofilm from normal flora on the mouse skin. As we showed in our previous work, the presence of both bacterial biofilm and high levels of OS are necessary and sufficient to initiate the chronicity of wounds; eliminating either bacterial biofilm or high levels of OS results in wound closure [13,14]. To investigate the role of different cell types in the initiation of chronicity, single-cell RNA sequencing of the NCWs and CWs was performed. To ensure the integrity of the RNA, we developed a robust protocol enabling us to isolate single cells from digested skin tissue in a very short period of time. Single-cell RNA sequencing (scRNA-seq) was performed on cells isolated from NCWs and CWs within 72 h post-wounding. Our findings show that the proportion of cell composition of wounds that heal is different from that of wounds that become chronic, even at early timepoints after injury. These differ not only in the number of the different cell types but also differ in their gene expression profile that alter cellular processes between the two conditions and contribute to the initiation of chronicity. These findings can potentially be used to guide further studies that inform therapeutic targets in CW care.

## 2. Materials and Methods

### 2.1. Compliance

All experiments were completed in accordance and compliance with federal regulations and the University of California policy and procedures were approved by the Institutional Animal Care and Use Committee (IACUC) under the animal use protocol (AUP) #11 at University of California, Riverside.

### 2.2. Reagents

MACS solution (Miltenyi Biotec, Auburn, CA, USA; cat no. 130-100-008); Whole Skin Dissociation Kit (Miltenyi Biotec; cat no. 131-101-540) that contains Buffer L, Enzyme P, Enzyme D, and Enzyme A; 1X Phosphate Buffer Saline; Trypan blue; 1X DMEM (Dulbecco’s Modified Eagle Medium) (Gibco, Waltham, MA, USA; cat no. 11995-065). 3-amino-1, 2, 4-triazole (ATZ, Tokyo Chemical Industry, Tokyo, Japan, cat no. A0432) and mercaptosuccinic acid (MSA, Thermo Scientific Chemicals, Waltham, MA, USA, B23301.22) to inhibit antioxidant enzymes. Evercode Cell Fixation v2 (Parse Biosciences, Seattle, WA, USA) fixation kit (SKU: ECF2001) for single-cell fixation. Parse Biosciences Evercode™ WT Mini kit (SKU: ECW02030, Parse Biosciences, Seattle, WA, USA) for library preparation. NextSeq 2000 P3 XLEAP-SBS Reagent Kit (300 cycles, 2 × 150 bp, Illumina cat no. 20100988, Illumina, San Diego, CA, USA) for sequencing.

### 2.3. Collection of Skin Tissues

The description of how to obtain chronic wounds in *db/db* mice has been published in detail by us previously [11,13,14]. Briefly, *db/db* mice were bred in our conventional vivarium from B6.BKS(D)-*Lepr^db^/J* heterozygotes purchased from the Jackson Laboratories (Bar Harbor, ME, USA; Stock no. 000697). Only *db/db* mice that were at least 5 months old and weighed 50 g or more were used to create CWs to allow the diabetes sufficient time to affect physiology, much like in humans and because the burden of the chronic wound causes the mouse to lose a significant amount of weight. To induce wound chronicity, diabetic mice were injected intraperitoneally with 3-amino-1, 2, 4-triazole (ATZ) 20 min before wounding to inhibit catalase and treated with mercaptosuccinic acid (MSA) locally on top of the wound, immediately after wounding, to inhibit glutathione peroxidase. Diabetic mice in which the wounds are not treated with ATZ and MSA are designated as NCW. We made 7 mm full-thickness cutaneous wounds on the back of the mice anesthetized with isoflurane. Tissue within 2 mm of the edges of the wounds was collected from different mice at 12, 24, 48, and 72 h after surgery, and the four samples combined for the analysis to obtain a view of what is occurring during the first 72 h post-injury in CWs versus NCWs.

### 2.4. Digesting the Full-Thickness Skin Wound Tissues (Figure 2)

An amount of 400 mg of fresh wound skin tissue was weighed, rinsed in 2 mL MACS solution for 30 s to remove excess blood, placed in a sterile Petri dish, and thoroughly minced with straight, fine-pointed scissors before being transferred into a sterile 1.5 mL microcentrifuge tube. Excess red blood cells that were trapped in the minced tissue were removed by washing several times with 1 mL of MACS solution and vortexed to dislodge any additional red blood cells trapped in the tissue. The minced tissue was centrifuged briefly in a tabletop zip spinner to separate the red blood cells from the minced tissue and the supernatant was removed. The procedure was repeated until the tissue was no longer releasing red blood cells. We prepared aliquots of Enzyme P, reconstituted the lyophilized powder of Enzyme D in the vial with 3 mL of Buffer L supplied with the kit, and reconstituted the lyophilized powder of Enzyme A in the vial with 1 mL of Buffer L, also supplied with the kit. Aliquots of all three enzymes were stored at −20 °C. We then added 435 µL of Buffer L and 12.5 µL of Enzyme P into the tube containing the minced tissue and mixed carefully with a spatula. Amounts of 50 µL of Enzyme D and 2.5 µL of Enzyme A were premixed and added to the tube and mixed carefully by gently vortexing the mixture or manually mixing with a spatula. The minced tissues were always fully submerged in the mixture. The 1.5 mL microcentrifuge tubes with skin samples and enzymes were placed into a 37 °C water bath for 2.5–3 h. Every 15 min for the first 2 h, the samples were briefly vortexed to disperse the minced tissue in the enzyme solution. Samples were gently centrifuged to bring down skin fragments stuck on the top and side walls of the tube. Skin fragments remained intact for the first 1–1.5 h and started to become stringy as the enzymes broke down the ECM. After 2.5 h, the tissue mixture was passed multiple times through an 18 G needle on a sterile 5 mL tube to separate cells without destroying them.

**Figure 2:**
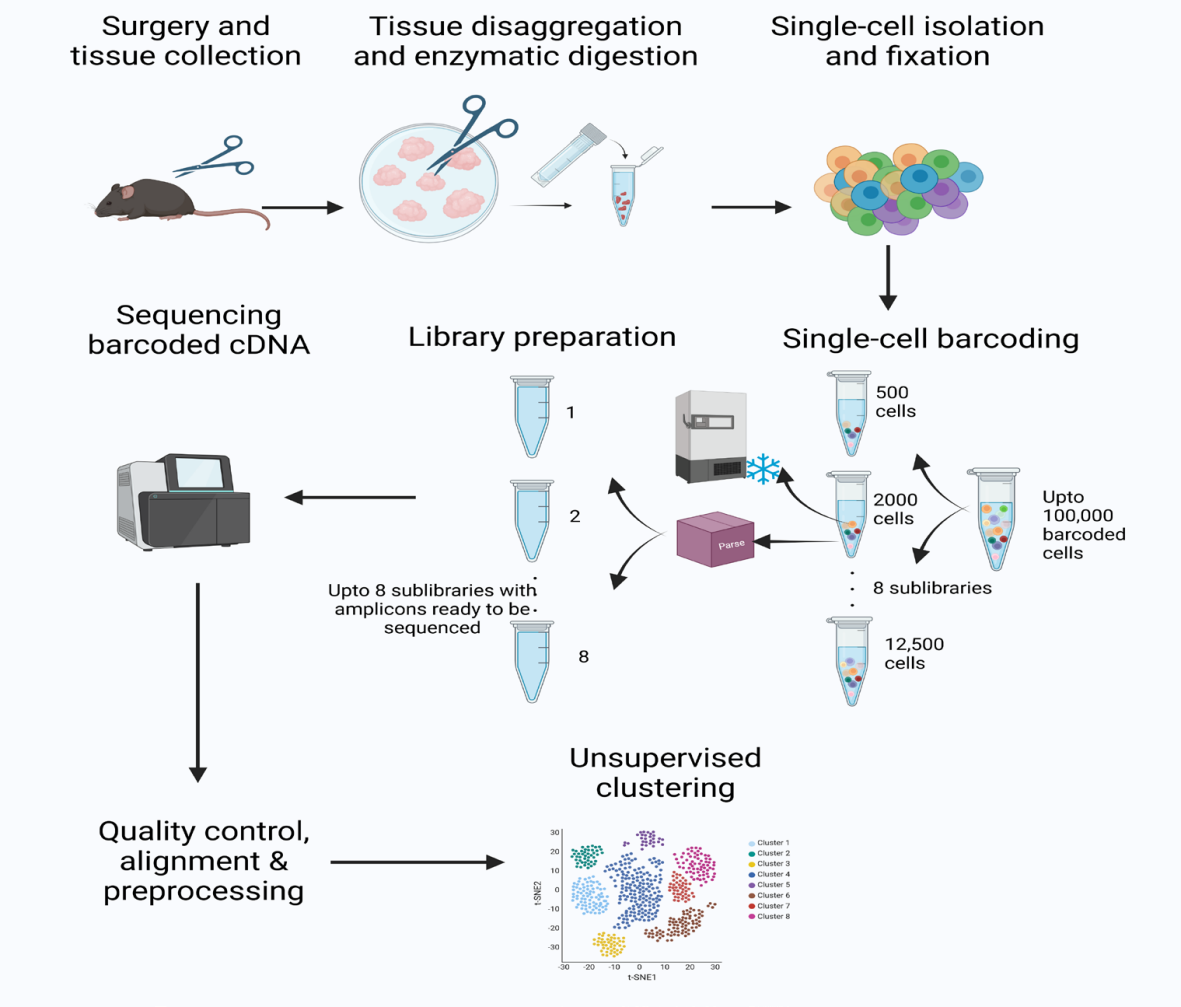
Schematic representation of the processes followed to perform single-cell RNA sequencing (scRNAseq) during initiation of wound chronicity. Full-thickness wounds were created on the back of the db/db mice under anesthesia. Chronic wounds (CWs) were treated with inhibitors for catalase and glutathione peroxidase to increase oxidative stress (OS) in the wound, with non-chronic wounds (NCWs) as control, healing wounds. Tissue specimens were collected from the edge of the wounds within 72 h post-surgery. Single cells were isolated, fixed, barcoded, and sequenced. Expression profiles of the single cells were used to obtain clusters using Uniform Manifold Approximation and Projection (UMAP).

### 2.5. Obtaining a Single Cell Suspension (Figure 2)

Once the ECM of the tissue samples was thoroughly disrupted, the cell suspension mixture was strained through a 40 µm cell filter into a 15 mL falcon tube, previously blocked with bovine serum albumin (BSA). The cell suspension was centrifuged at 300 RCF for 15 min at 4 °C and the supernatant was carefully removed without disturbing the cell pellet at the bottom of the tube. The cell pellet was carefully resuspended in 4 mL of cold DMEM media, strained again through another 40 µm filter, centrifuged at 300 RCF for 15 min at 4 °C before the supernatant was removed. The cell pellet was carefully resuspended with 2 mL of cold DMEM media before counting cells. To count the number of live single cells, 10 µL of Trypan blue was slowly and thoroughly mixed with 10 µL of the cell suspension in a 1.5 mL microcentrifuge tube. An amount of 10 µL of the Trypan blue and cell suspension mixture were added to a hemocytometer and the living cells were counted; dead cells stained blue whereas live cells did not.

### 2.6. Fixation of the Cells and Barcoding (Figure 2)

The Parse Biosciences Evercode WT was used for fixation and barcoding of the RNA. This assay uses a split and pool approach to generate a unique combination of barcodes, labeling the transcriptome of individual cells in a sample of up to a million cells [15]. For all samples, the single cells were fixed using the Evercode Cell Fixation v2 Parse Biosciences fixation kit (SKU: ECF2001), and cryopreserved until all tissues were collected. Samples were all run together to minimize batch effects. As the cells were permeabilized but remained intact, this effectively rendered the individual cells as a reaction vessel. The barcode reactions appended three different barcodes to each transcript within the cell. ScRNA-seq libraries were prepared using the Parse Biosciences Evercode™ WT Mini kit (SKU: ECW02030). Briefly, samples were normalized by cell number and added to the well(s) of a 96-well plate to perform reverse transcription, adding a well-specific barcode (first barcode). All wells were then pooled and randomly distributed across a new 96-well plate with new well-specific barcodes (second barcode) for ligation. After ligation of the second barcode, wells were again pooled and randomly re-distributed across another 96-well plate for a third round of barcoding. The cells were then pooled into sub-libraries with a fourth, sub-library-specific barcode added. Then, the cells were lysed for the construction of the final cDNA libraries, and sequencing. This scRNA-seq approach effectively removed ribosomal RNA contamination as the odds of a free-floating ambient RNA molecule traveling the same path as a cell through 4 rounds of splitting, barcoding, and pooling are 1 in 3,538,944. In addition, to make Illumina sequencing more efficient, the workflow involved a wash step that physically removed free-floating molecules from barcoded cells before they were lysed and processed for sequencing, further reducing the chances that any environmental RNA molecule would be incorrectly grouped with a cell in downstream analyses. The Parse Biosciences Evercode™ WT Mini kit (SKU: ECW02030) generates libraries of an average of 450 bp. The libraries were sequenced on an Illumina NextSeq 2000 sequencer using a NextSeq 2000 P3 XLEAP-SBS Reagent Kit (300 cycles, 2 × 150 bp, paired-end sequencing at 150 bp read length, Illumina cat no. 20100988). Data were demultiplexed into sets of sub-library FASTQ files that were processed using Parse Bioscience’s analysis pipeline. The output was a set of html summary files that provided an overview of the sequencing quality control (QC) and a digital gene count matrix.

### 2.7. Single-Cell Data Processing and Quality Control (QC)

Raw data were pre-processed using the *splitpipe* (v1.0.3) (https://www.parsebiosciences.com (accessed on 26 January 2023) tool from Parse Biosciences. Specifically, the pipeline parsed the combinatorial barcodes for each library with parameters ‘split-pipe—mode all’ and aligned the reads against the mouse reference genome (GRCm39/mm10) using STAR aligner (2.7.10b) [16]. The pipeline output digital gene expression matrices contained “filtered” or “unfiltered” data for the single-cell whole transcription experiment. The gene expression matrix containing “unfiltered” data was imported into R (v4.2.2) (R Core Team 2022) with the Seurat (v4.3) [17] package using the convenience function *ReadParseBio*. QC analysis was carried out on the “unfiltered” data to identify and remove low-quality cells. Specifically, cells were initially selected for further downstream analysis using the following criteria: gene counts > 100, unique molecular identifier (UMI) counts per cell > 500 and <50,000, and percent mitochondrial genes < 15%. The filtered cells were further subjected to doublet detection using *scDblFinder* [18] with default settings.

### 2.8. Normalization, Dimensionality Reduction, and Clustering

The final dataset containing cells quality-controlled for mitochondrial genes, UMIs, gene counts, and doublet exclusion was used for normalization, linear (PCA), and non-linear (*t*-SNE, UMAP) dimension reduction and clustering analysis within the *Seurat* package. Data were normalized using the Seurat *SCTransform* (v0.3.5) package [19,20] with method “*glmGamPoi*” and regression of the percentage of mitochondrial genes mapping. Principal component analysis (PCA) within the Seurat package was performed using *RunPCA* with parameter ‘npcs = 30’. Cell clusters were identified using the principal components from *RunPCA* with the *FindNeighbors* and *FindClusters* functions within Seurat. Resolution used for *FindClusters* was 0.3. Cluster marker identification was carried out using the Seurat *FindAllMarkers* function with the Wilcox likelihood ratio test and Bonferroni-corrected *p*-value. Markers were defined as genes with expression in more than 25% of the cells within the cluster and having a positive logarithmic base 2-fold change (log2FC) > 0.25 against all other clusters and an adjusted *p*-value < 0.05.

### 2.9. Subclustering Analysis

To identify subclusters within the annotated cell clusters (e.g., fibroblasts), we extracted cells belonging to designated clusters using the base R subset function on the aggregated normalized and filtered Seurat object. The reduced dataset (containing only selected cells) was subjected to the standard Seurat workflow for PCA dimensionality reduction and clustering using the same parameters and settings as above. *FindClusters* (v4.3) was used to obtain subclusters with resolution = 0.3. The number of subclusters was determined by the *FindClusters* function, not manually or arbitrarily.

### 2.10. Analysis of Differentially Expressed Genes and Annotation

To identify genes that were differentially expressed between NCWs and CWs, raw read counts were extracted from Seurat and analyzed for differentially expressed genes (DEGs) using *DESeq2* algorithm (version 1.40.2) [21]. Log2FC was shrunken by DESeq2 function *lfcShrink* (type = “*apeglm*”) to reduce noises to signal ratio for genes with low expression [22]. All analyses were performed in R (version 4.3.1). For comparisons between Fib 1 and Fib 2 cells in each of the wound conditions, strict criteria for differential expression analysis were set to avoid false discovery of genes by setting the log2FC ≥ 1 and an FDR < 0.0001. However, for the comparisons of each cluster between NCWs and CWs, less strict DEGs were defined with log2FC > 0.5 and FDR < 0.05. Gene Ontology (Biological Processes version 2020-09-16) enrichment analysis was carried out using Metascape (Version 3.5, http://metascape.org (accessed on 26 March 2024)). *p*-values were calculated using the cumulative hypergeometric distribution with the Benjamini–Hochberg correction method for multiple testing. Significantly overrepresented GO terms were defined as having an adjusted *p*-value < 0.01 and an enrichment factor > 1.5 with a minimum overlap > 3. A pathway was considered “enriched” if it was overrepresented in the list of upregulated genes and was considered “suppressed” if it was overrepresented in the list of downregulated genes.

### 2.11. Plots and Illustrations

Volcano plots and bar graphs were made with the *ggplot2* (v3.5.0) package in R. Illustrations were made in BioRender (http://biorender.com (accessed on 20 April 2024)). Heatmaps, violin plots, and UMAP plots were generated using the functions built in to *Seurat* (v4.3) and *scCustomize* (v1.1.3) [23].

## 3. Results

### 3.1. Skin Cell Types Identified by scRNA-Seq

To understand the roles of different cells in the initiation of wound chronicity, we used our mouse model of chronic wounds [11,13,14] to obtain tissue from NCWs and CWs, performed scRNA-seq analysis, and then compared the transcriptomic landscape of both wound conditions. We profiled a total of 102,737 cells from scRNA-seq, with 75,511 cells remaining after quality control and filtering (see the Methodology Section).

**Figure 3:**
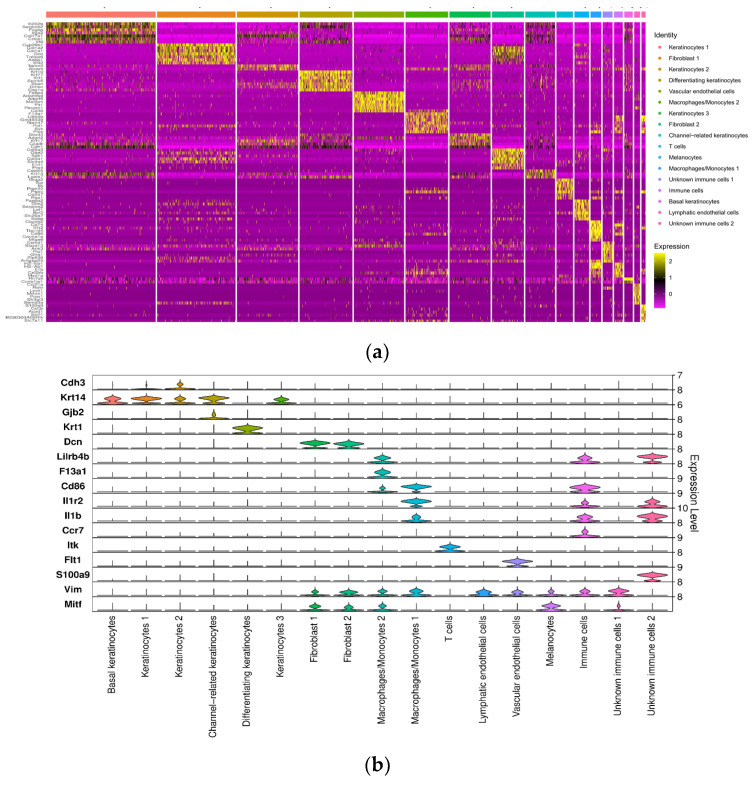

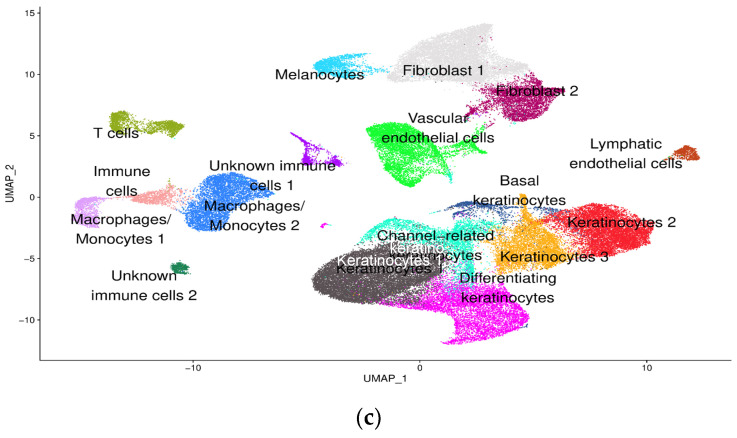
ScRNAseq allowed identification of all major cell types involved in healing. (**a**) Stacked violin plot of the hallmark genes used to annotate the obtained clusters. (**b**) Heatmap of top differentially expressed genes (DEGs) in each of the clusters. Yellow shows higher expression. (**c**) UMAP embedding of the final dataset consisting of 75,511 cells. The cells are colored by orthogonally generated clusters and labeled by manual cell type annotation. Seventeen clusters were obtained from UMAP classification of the single-cell expression profiles.

To annotate the clusters, we used a combination of the top-expressed genes and hallmark cell markers for different skin cell types (Figure 3a,b, Table 1). For keratinocytes, we used keratin expression as the anchor marker. Krt14 [24], a marker for basal keratinocytes, was expressed in several clusters, suggesting acquisition of basal keratinocyte characteristics by several keratinocytes during wound healing. We then used specific markers to distinguish the various clusters of keratinocytes: for Keratinocyte 1 (Kerat 1), Irf6 [25]; for Kerat 2, Dmkn [26], Krt17 [27], and Sbsn [28]; for Kerat 3, Cxadr [29]; and for Channel-related keratinocytes (Channel Kerats), Gjb2 [30]. Krt1 [24], a marker for the suprabasal differentiated keratinocytes, was expressed in differentiating keratymocites (Diff Kerats). For fibroblasts, we used Dcn [31] as the anchor gene and then distinguished the two clusters using Col1a1 for Fib 1 (pro-healing) and Col3a1 for Fib2 (non-healing). For vascular endothelial cells (VECs), we used Pecam1 [30] and Flt1 [31], and for lymphatic endothelial cells (LECs), Vim [32], Sema3a [33], and Prox1 [34]. For the immune cell types, we used CD86 [35] and Il1b [36], for proinflammatory macrophages (M1) and Lilrb4b [37] and F13a1, for anti-inflammatory macrophages (M2). For T cells, we used CD247 [38] and Rgs1 [39], and for unspecified immune cells, Scl7a11 [40], Csf3r [41], and S100a9 [42] (Figure 3a,b, Table 1). After annotation, we identified a total of 17 distinct cell clusters (Figure 3c).

**Figure 4:**
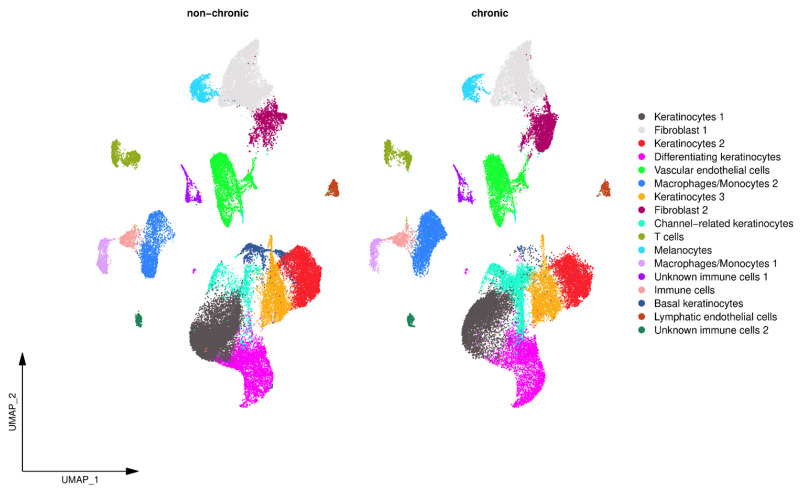
Comparison of the identified clusters between NCWs and CWs. Several clusters show differences in the number of cells between the two wound conditions. Of the two distinct fibroblast clusters, we observed a higher number of pro-healing fibroblasts (Fib 1) in NCWs whereas non-healing fibroblasts (Fib 2) were more abundant in CWs. Of the clusters identified as keratinocytes, Kerat 1 and Basal Kerats were more abundant in NCWs, Kerats 2, 3, and Diff Kerats showed no difference, whereas Channel Kerats were considerably more abundant in CWs. VECs and LECs were more abundant in NCWs. Several clusters were identified to be immune cells. Proinflammatory macrophages (M1) were more abundant in NCWs, and anti-inflammatory macrophages (M2) were more abundant in CWs.

A comparison of the identified clusters between NCWs and CWs revealed substantial differences in the number of cells in several of the clusters (Figure 4, Table 2). Of the two distinct fibroblast clusters, we observed a higher number of Fib 1 in NCWs whereas Fib 2 was more abundant in CWs. Of the clusters identified as keratinocytes, Kerat 1 and Basal Kerats were more abundant in NCWs, Kerats 2, 3, and Diff Kerats showed no difference, whereas Channel Kerats were considerably more abundant in CWs. Among endothelial cells, the majority of the cells were VECs rather than LECs and both endothelial cell types were more abundant in NCWs. Several clusters were identified to be immune cells, and of these, some clusters could be annotated with specific immune cell types, such as T cells and M1 and M2 (Figure 4, Table 2). The majority of the immune cell clusters showed higher numbers of cells in NCWs than in CWs, with the exception of M2 macrophages, which were much more abundant in CWs.

### 3.2. Fibroblast 2 Shows Altered Cellular Processes That Contribute to Chronicity

Fibroblasts are among the most important cell types during wound healing. At the early stages of the healing process, they migrate toward the wound, proliferate, secrete pro-healing cytokines, and synthesize ECM components, all of which contribute to the formation of the granulation tissue. At later stages of healing, they differentiate into myofibroblasts that contract to reduce the wound area [5].

**Figure 5:**
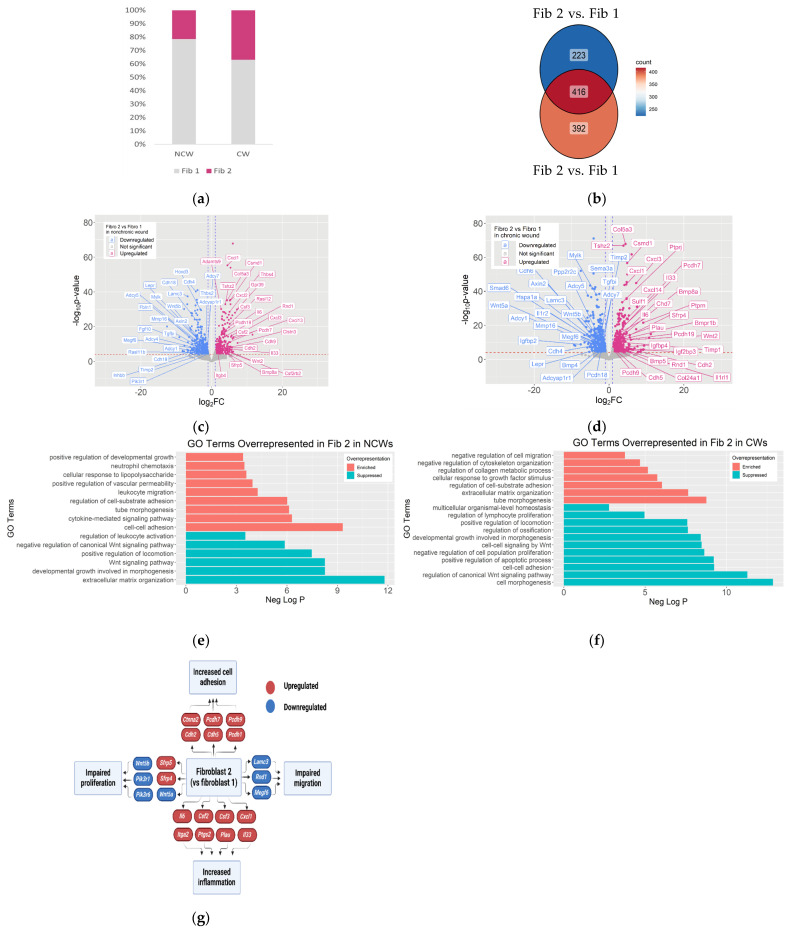
Fibroblasts enriched in CWs show disruption of several pro-healing mechanisms. (**a**) Stacked bar plots showing the proportion (*y*-axis) of Fib 1 and Fib 2 cells in NCWs and CWs. (**b**) Venn diagram showing DEGs between Fib 1 and Fib 2 in NCWs and CWs. A total of 416 genes were common between the two comparisons. (**c**) Volcano plot showing DEGs between Fib 1 and Fib 2 in NCWs. Pink dots show genes upregulated in Fib 2 and blue dots genes downregulated in Fib 2. Because two different clusters were compared, we used more stringent conditions: FDR < 0.0001 and Log2FC > 1 in absolute value. (**d**) Volcano plot showing genes differentially expressed between Fib 1 and Fib 2 in CWs. The same parameters as in (**c**) were applied. (**e**) Selected biological pathways that are significantly (*p*-value < 0.01) affected in the comparison of Fib 2 and Fib 1 cells in NCWs. The directionality of overrepresentation of each pathway is depicted using a pseudo color (orange/rusty for enriched, blue/green for suppressed). Significantly overrepresented GO terms were defined as having an adjusted *p*-value < 0.01 and an enrichment factor > 1.5 with a minimum overlap >3. A pathway was considered “enriched” if it was overrepresented in the list of upregulated genes and was considered “suppressed” if it was overrepresented in the list of downregulated genes. The pathway analysis was performed in Metascape using Gene Ontology terms. A term was considered overrepresented when *p* <  0.01, had a minimum count of 3, and an enrichment factor > 1.5, which is the ratio between the observed counts and the counts expected by chance. (**f**) Selected biological pathways that are significantly (*p*-value < 0.01) affected in the comparison of Fib 2 and Fib 1 cells in CWs. (**g**) Cellular processes involved in healing that are affected by DEGs between Fib 1 and Fib 2.

Of the two clusters identified as fibroblasts, Fib 1 was more abundant in NCWs whereas Fib 2 was more abundant in CWs (Figure 5a, Table 2). To understand the inherent differences between these fibroblasts, gene expression profiles of the two clusters were compared within each wound condition with strict criteria used for these analyses by setting the FDR < 0.0001. We also set the Log2FC > 1 in absolute value. A total of 639 genes were differentially expressed between Fib 1 and Fib 2 in NCWs and a total of 808 genes were differentially expressed between Fib 1 and Fib 2 in CWs. A total of 416 of these DEGs were common between the two comparisons, suggesting that these common genes are very central to fibroblast function in general (Figure 5b). Differential gene expression (DEG) analysis of Fib 1 and Fib 2 showed that Fib 2 in NCWs had 279 upregulated genes and 360 downregulated genes, some of which have been annotated on a volcano plot (Figure 5c, Table S1). A similar analysis performed to compare Fib 1 and Fib 2 in CWs showed 357 upregulated genes and 451 downregulated genes in Fib 2, also annotated on a volcano plot (Figure 5d, Table S2).

Pathway analysis of the DEGs between Fib 1 and Fib 2 in NCWs and in CWs showed that several pathways leading to cell proliferation or migration were disrupted in Fib 2 (Figure 5e–g). One such pathway negatively affected in Fib 2 cells is the Wnt signaling, which several studies have shown its importance for pro-healing mechanism [43,44]. Despite upregulation of Wnt2 in these cells, Wnt signaling was dysfunctional because of the downregulation of Wnt5a and Wnt5b as well as upregulation of Sfrp4 and Sfrp5, which inhibit Wnt signaling by blocking frizzled receptors. Downregulation of PI3K/Akt signaling (Pik3r1, Pik3r6, and Pik3cb) can also interfere with several pro-healing mechanisms in Fib 2, including proliferation and cell migration [45]. Proper proliferation of fibroblasts is important to form granulation tissue by providing a constant pool of fibroblasts [46].

Our data show enrichment of cell–cell adhesion molecules such as Cadherin signaling (Ctnna2, Cdh2, Cdh5, Pcdh7, Pcdh9, and Pcdh19) that when coupled to the suppression of Integrins signaling (Lamc3, Rnd1, and Megf6) and TGF-beta signaling (Smad6, Bmp8a, Bmp5, Bmp4, and Bmper) can interfere with the ability of Fib 2 to migrate [46,47,48]. We also show that Fib 2 have downregulated Mmp11 and Mmp16, and upregulated levels of Timp1. Fibroblasts migrate by adhering to the components of the ECM to move toward the center of the wound and contribute to the formation of the granulation tissue [49]. Therefore, temporal regulation of the expression and activation of metalloproteases (MMPs) and tissue inhibitors of metalloproteases (TIMPs) is crucial for wound healing as they can alter the ECM composition required for cell migration [50,51].

cAMP-dependent signaling is also negatively affected in Fib 2 through downregulation of several members of the adenylate cyclase family, such as Adcy1, Adcy5, Adcy7, and Adcyap1r11. cAMP-dependent signaling plays fundamental roles in several cellular functions including cell migration and proliferation, and downregulation of genes in this signaling pathway can interfere with the development of the granulation tissue [52,53,54]. Furthermore, upregulation of several tumor suppressor genes in Fib 2, such as Csmd1, Sulf11, Ptprj, and Tshz2, can interfere with cell proliferation in these cells [54,55,56]. In addition, the Jak/Stat signaling pathway is potentially affected in Fib 2 as Stat3 (log2FC-0.98) and Jun (log2FC-1.65) showed downregulation in these cells in CWs. Downregulation of these genes can negatively impact cell migration and proliferation, interfering with the formation of granulation tissue [57].

It is well known that moderate levels of reactive oxygen species (ROS) and inflammation are required for healing [58]. However, CWs have high levels of OS and chronic inflammation [59]. Fib 2 shows upregulation of several genes that lead to increased inflammation. Of these genes, Il1rl1, Il6, Cxcl1, Cxcl2, Cxcl3, Cxcl5, Cxcl13, Cxcl14, Csf2, Csf3, Ptgs2, Itga2, Plau, and Rgs4 were upregulated. Increased expression of proinflammatory cytokines in CWs suggests the importance of higher levels of inflammation in the initiation of chronic wounds [60,61,62]. In addition, upregulation of Il33 in Fib 2 promotes the activation of monocytes into M2 macrophages, which could explain the higher number of these macrophages in CWs [63]. Whereas M2 macrophages at later stages of healing are required for the formation of the granulation tissue and scavenging of cellular debris [64], premature activation of these macrophages can blunt the acute inflammatory response required for normal wound healing [65]. The findings that M2 macrophages are more abundant in non-healing conditions are counter-intuitive.

In addition, Fib 2 can negatively impact phagocytosis in CWs. These fibroblasts show downregulation of leptin receptor (Lepr), which decreases the production of monocyte chemoattractant proteins that in turn decreases the recruitment of monocytes to the wound microenvironment at the early stages of healing [66]. This results in fewer M1 macrophages that are important for the clearing of pathogens and cellular debris through phagocytosis, contributing to chronicity.

Whereas Fib 1 cells can be generally considered as pro-healing cells, Fib 2 cells show disrupted pro-healing functions. Given the higher number of cells in Fib 2 compared to Fib 1 in CWs, the detrimental effects of these fibroblasts can be observed more in CWs. These findings are similar to those reported in a study of human DFUs where non-healing wounds showed higher numbers of what the authors called non-healer fibroblasts [65].

**Figure 6:**
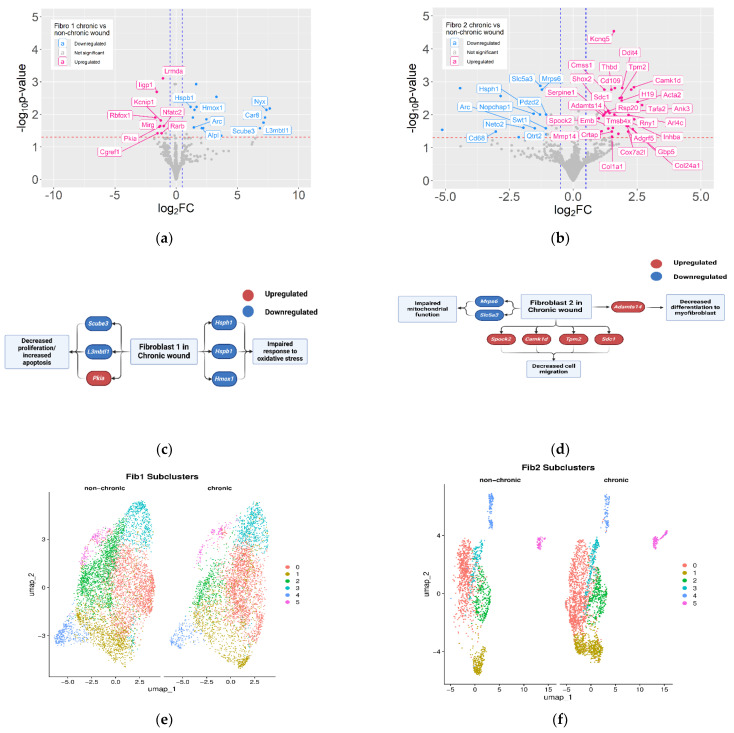
Wound conditions can affect the function of fibroblasts in response to injury. (**a**) Volcano plot showing genes differentially expressed in Fib 1 between NCWs and CWs. As the same clusters were compared in this analysis, less stringent criteria for differential expression were used; FDR < 0.05 and Log2FC > 0.5 in absolute value. Pink dots show genes upregulated in CWs and blue dots genes that are downregulated. (**b**) Volcano plot showing genes differentially expressed in Fib 2 between NCWs and CWs. Pink dots show genes upregulated in CWs and blue dots genes that are downregulated. (**c**) Cellular processes important for healing that are negatively affected in Fib 1 in CWs compared to NCWs. (**d**) Cellular processes important for healing that are negatively affected in Fib 2 in CWs compared to NCWs. (**e**) Fib 1 subclusters between NCWs and CWs. All subclusters were more abundant in NCWs, with the exception of subcluster 0, showing 4% decrease in NCWs. (**f**) Fib 2 subclusters between NCWs and CWs. All subclusters showed higher cell numbers in CWs, with the exception of subcluster 4, showing 39% decrease in CWs.

We also compared the expression profile of Fib 1and Fib2 between NCWs and CWs to analyze the effects of the wound microenvironment on their gene expression profile. As the same clusters were compared in this analysis, less stringent criteria for differential expression were used. We set the FDR < 0.05 and the Log2FC) > 0.5 in absolute value. Whereas the list of DEGs was not as extensive as in the comparison of Fib 1 and Fib 2 within NCWs and within CWs, this analysis pointed to substantial differences in each fibroblast type in the two wound conditions (Figure 6a,b, Tables S3 and S4), which could affect several of the cellular processes involved in wound healing (Figure 6c,d).

Fib 1 in CWs showed downregulation of Hmox1, which plays an important role in degrading heme in the wound microenvironment. Downregulation of Hmox1 in CWs can lead to persistently high levels of heme, which can contribute to the high levels of OS [67]. In addition, Fib 1 may be more susceptible to OS in CWs due to downregulation of the heat shock gene, Hspb1. During normal wound healing, expression of heat shock proteins (HSPs) is increased in response to injury [68]. Downregulation of these proteins in CWs can contribute to the initiation of chronicity. Fib 1 in CWs did not express L3mbtl1, which plays a role in promoting mitosis, therefore potentially contributing to the lower number of Fib 1 in the CWs [69]. Downregulation of Scube3, a regulator of BMP signaling [70], can contribute to the suppression of cell migration in CWs, a cornerstone of the formation of granulation tissue. In addition, Alpl, the gene encoding alkaline phosphatase, a potential marker for wound healing [71], was downregulated in Fib 1 in CWs. Differential expression of these genes involved in cell proliferation may explain the lower number of Fib 1 in CWs.

Comparison of Fib 2 between NCWs and CWs showed that several genes involved in ECM organization were abnormally expressed. Upregulation of Mmp14 in Fib 2 in CWs can lead to degradation of the ECM [72]. Previous studies have shown that MMP-14 is associated with chronic wounds [73]. This MMP can degrade both collagen I and III [73]. Collagen III is the main type of collagen in the ECM during the early stages of wound healing. Degradation of this collagen by MMP-14 can contribute to non-healing. Upregulation of Camk1d, Spock2 [74,75], Ank3 [76], Tpm2 [77], and Sdc1 [78] in Fib 2 in CWs can negatively affect cell migration by slowing down focal adhesion disassembly [79]. In addition, downregulation of mitochondrial Mrps6 suggests impaired mitochondrial function of Fib 2 in CWs [80]. Whereas the role of mitochondria ribosomal protein S6 (MRSP6) in wound chronicity has not been studied, its role in mitochondrial oxidative phosphorylation (OXPHOS) and inflammatory diseases has been investigated in humans [81,82]. Proper mitochondrial OXPHOS is required for providing energy to the highly dynamic pool of cells in the wound microenvironment [82]. Fib 2 in CWs also showed upregulation of Cox7a21, another gene involved in mitochondrial OXPHOS [83], providing further evidence on the role of dysfunctional mitochondria in the initiation of chronicity. Upregulation of Adamts14 in Fib 2 in CWs can negatively affect myofibroblast differentiation [84]. Myofibroblasts play a fundamental role in wound closure as they contract to bring the edges of the wounds closer together [5]. Upregulation of several genes that are involved in cell proliferation, including Inhba [85], Tafa2 [86], Rps20 [87], Ddit4 [88], and Sdc1 [79] in Fib 2 in CWs, could explain higher numbers of Fib 2 in CWs. This imbalance between Fib 1, which promotes healing, and Fib 2, which can interfere with healing by increasing inflammation and impairing processes involved in proliferation and migration, coupled with a lower number of Fib 1 and higher numbers of Fib 2 in CWs, can contribute to the initiation of the wound chronicity from early stages after injury.

To better understand how Fib 1 and Fib 2 may contribute to wound healing or chronicity, these clusters were subclustered (Figure 6e,f). Of the six subclusters of Fib 1, subclusters 2 and 4 showed the most difference in cell numbers between NCWs and CWs, being 3.4-fold and 1.84-fold lower in CWs, respectively. Differential expression analysis of these clusters between NCWs and CWs with FDR < 0.05 and log2FC > 0.5 in absolute value was performed. Fib 1 subcluster 2 showed downregulation of Hmox1, which can increase the OS in CWs. This subcluster also showed downregulation of Bmp7 and Tnc, which can suppress the migration of cells in this subcluster [89,90]. Subcluster 4 of Fib 1 showed downregulation of Hsp90aa1, which disrupts the response of this subcluster of cells to OS. In addition, this subcluster of Fib 1 showed upregulation of Icam1 and Camk1d, which can inhibit migration of these cells [91,92,93].

All Fib 2 subclusters showed remarkable differences in the number of cells between NCWs and CWs. In CWs, various subclusters showed an increase in cell numbers: subcluster 0, 1.59-fold, subcluster 1, 2.26-fold, subcluster 2, 1.34-fold, subcluster 3, 1.51-fold, and subcluster 5, 2.14-fold. Subcluster 4 was an exception to this trend with a 0.6-fold decrease in cell numbers in CWs. Differential expression analysis of these subclusters showed that several of the detrimental processes observed in Fib 2 in CWs are also observed in some of the subclusters, even though two of these subclusters did not show differentially expressed genes between NCWs and CWs. Differential expressions of several genes in Fib 2 subcluster 0 could affect cellular functions fundamental for healing, such as proliferation, migration, and mitochondrial function. Downregulation of Lrrk1 and upregulation of Ndrg1 and Sdc1 in CWs can negatively affect migration of these cells in CWs [79,94,95]. Downregulation of Sesn2 and upregulation of Ddit4 in CWs can negatively impact cell proliferation [96,97]. Fib 2 subcluster 0 also showed downregulation of Mrsp6 and upregulation of Cox5a and Fabp5, suggesting changes in cell metabolism and mitochondrial function in these cells in CWs. Whereas upregulation of Cox5a may protect mitochondria from OS [98], it is not clear whether this translates to better overall mitochondrial function as other mitochondrial components such as Mrsp6 are downregulated in this subcluster. This subcluster also shows downregulation of Hsph1, which can negatively impact the cellular response to OS. In addition, upregulation of Mmp14 and Timp1 in CWs in Fib 2 subcluster 0 suggests changes in the composition of the ECM. Fib 2 subcluster 4 showed upregulation of Camk1d in CWs, which can suppress migration of these cells in CWs [93]. Given that other subclusters in Fib 2 did not show DEGs between the two types of wounds, the subclusters with altered pro-healing functions could be driving chronicity.

### 3.3. Keratinocytes Show a High Level of Heterogeneity During Chronic Wound Initiation

**Figure 7:**
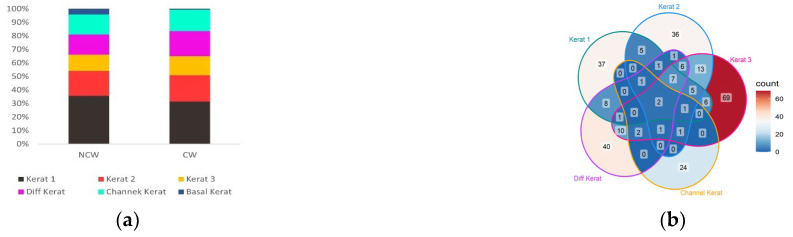

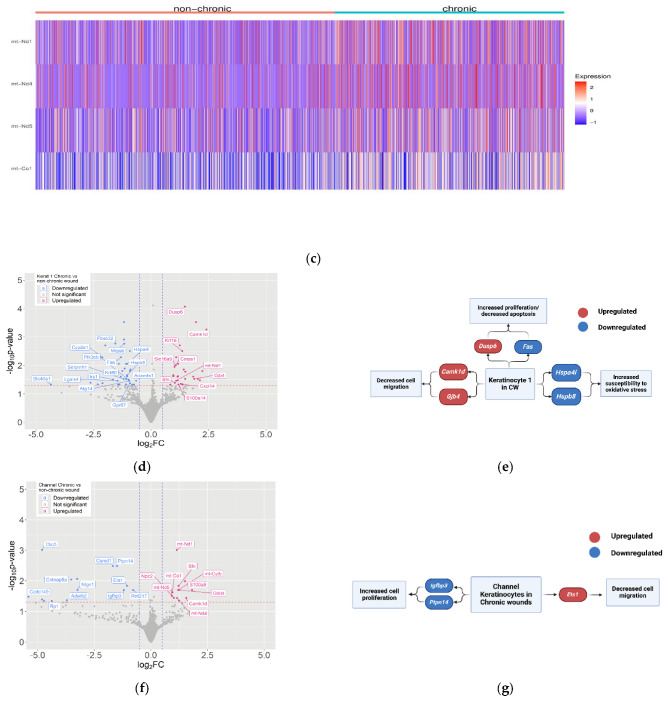
Keratinocytes have impaired healing mechanisms in response to injury in CWs. (**a**) Stack plot representing relative abundance of different subtypes of keratinocytes in NCWs and CWs. Most keratinocyte subtypes were more abundant in NCWs, except for Channel Kerats, suggesting potential roles of this keratinocyte in chronicity. (**b**) Venn diagram representing the number of DEGs in each keratinocyte type and their overlap between keratinocyte subtypes. (**c**) Heatmap showing genes involved in electron transport chain that are differentially expressed between NCWs and CWs. Relative gene expression is shown in pseudo color, where blue represents downregulation and red represents upregulation. (**d**) Volcano plot showing genes differentially expressed in Kerat 1 between NCWs and CWs. FDR < 0.05 and Log2FC > 0.5 in absolute value. Pink dots show genes upregulated in CWs and blue dots genes that are downregulated. (**e**) Cellular processes important for healing that are negatively affected in Kerat 1 cells in CWs compared to NCWs. (**f**) Volcano plot showing genes differentially expressed in Channel Kerats between NCWs and CWs. Pink dots show genes upregulated in CWs and blue dots genes that are downregulated. (**g**) Cellular processes important for healing that are negatively affected in Channel-related keratinocytes in CWs compared to NCWs.

Keratinocytes are the major cell type in the epidermis, and much like fibroblasts, they respond to proliferation signals during the inflammatory phase. Proliferation, migration, and maturation of keratinocytes result in proper re-epithelialization and re-establishment of the skin barrier. In the early stages post-injury, keratinocytes show a high level of diversity, as suggested by the six keratinocyte clusters identified in our analysis. Kerat 1 and Basal Kerats are more abundant in NCWs whereas Kerat 2, 3, and Differentiating Kerats do not show a substantial difference in numbers between NCWs and CWs. Conversely, Channel Kerats are more abundant in CWs, and Basal Kerats are virtually non-existent in these wounds (Figure 7a).

To understand the role of keratinocytes in wound healing beyond the cell populations, gene expression profiles of each subtype of keratinocyte were compared between the NCWs and CWs with FDR < 0.05 and Log2FC > 0.5 in absolute value. Several genes were found to be differentially expressed between the two wound conditions (Figure 7b). Interestingly, Kerat 1, Kerat 2, Kerat 3, and Channel Kerats in CWs showed upregulation of genes involved in mitochondrial oxidative phosphorylation. Examples of these genes are mt-Nd1, mt-Nd4, mt-Nd5, and mt-Co1 (Figure 7c). Upregulation of these genes in CWs can lead to increased production of ROS during mitochondrial respiration, which in turn contributes to high levels of OS in CWs [99].

Comparing the expression profile of Kerat 1 between NCWs and CWs showed differential expression of genes involved in cell proliferation, migration, and apoptosis (Figure 7d,e, Table S5). Downregulation of chaperon proteins Hspa4l and Hspb8 can render these cells more susceptible to the high levels of OS in CWs and dismantle the cellular processes required for re-epithelialization. Upregulation of Dusp6 in Kerat 1 in CWs can lead to increased proliferation of these cells [100]. Furthermore, Kerat 1 cells show downregulation of Fas in CWs, which decreases apoptosis in these cells. On the other hand, upregulation of Gjb4 and Camk1d in Kerat 1 in CWs can suppress migration of these cells toward the center of the wound [93]. This higher level of proliferation and decreased apoptosis along with decreased cell migration could explain the accumulation of keratinocytes at the edge of chronic wounds and the lack of wound coverage [101]. Kerat 1 in CWs also showed upregulation of Krt16. Krt16 was previously found to be increased in response to injury and can increase the expression of proinflammatory cytokines, which in turn increases the inflammation in CWs by recruiting more immune cells [102]. Also, Kerat 1 cells in CWs show higher expression of Cxcl14, which can further contribute to the high levels of inflammation associated with CWs by recruiting more immune cells [103].

Comparison of the expression profile of Channel Kerats in NCWs and CWs showed various pro-healing processes to be negatively affected in these cells in CWs (Figure 7f,g, Table S6). Channel Kerats in CWs showed downregulation of Igfbp3. This gene has previously been found to inhibit the proliferation of various cell types in an insulin-like growth factor (IGF)-independent manner [104]. Therefore, its downregulation in Channel Kerats in CWs can contribute to the higher number of these cells in these wounds. In addition, Channel Kerats showed downregulation of Ptpn14 in CWs. Ptpn14 has been shown to inhibit Yes-associated protein (YAP) transcriptional activity in the Hippo signaling pathway. Given the importance of Hippo signaling in promoting cell proliferation, downregulation of Ptpn14 can contribute to the increase in the number of Channel Kerats in CWs [105]. High expression of Lgals7 in Channel Kerats can potentially suppress migration due to increased cell–cell adhesion. In addition, these cells show downregulation of Ets1 in CWs, which can further negatively affect their migratory functions [106]. Even though the role of Channel Kerats has not been studied in the context of wound healing, given their gene expression profile in NCWs and CWs, these cells may contribute to the development of wound chronicity.

### 3.4. Lymphatic Drainage and Blood Circulation Are Dysfunctional in CWs

**Figure 8:**
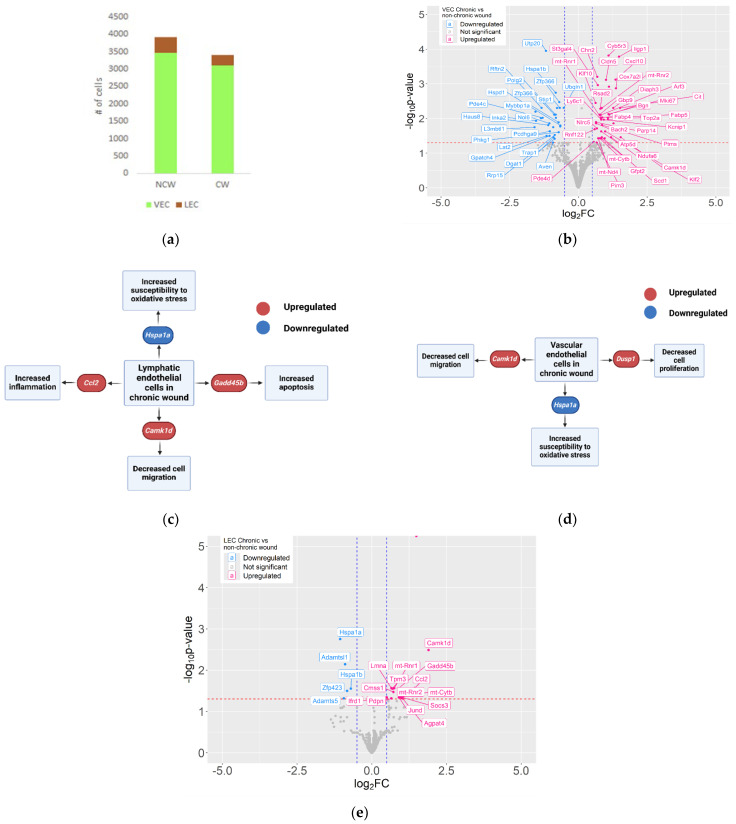
Endothelial cells show lower numbers and impaired healing functions in CWs. (**a**) Bar plots showing the number of cells (*y*-axis) of VECs and LECs in NCWs and CWs. (**b**) Volcano plot showing genes differentially expressed in VECs between NCWs and CWs. FDR < 0.05 and Log2FC > 0.5 in absolute value. Pink dots show genes upregulated in CWs and blue dots genes that are downregulated. (**c**) Volcano plot showing genes differentially expressed in LECs between NCWs and CWs. Pink dots show genes upregulated in CWs and blue dots genes that are downregulated. (**d**) Cellular processes important for healing that are negatively affected in VECs in CWs compared to NCWs. (**e**) Cellular processes important for healing that are negatively affected in LECs in CWs compared to NCWs.

The process of neovascularization starts shortly after injury in response to growth factors released during the early stages of healing. Neovascularization is critical for proper wound healing as new microvessels form in the granulation tissue, allowing the delivery of oxygen and nutrients to the highly proliferative populations of cells in the wound [5]. A comparison of NCWs and CWs showed lower numbers of VECs and LECs in CWs (Figure 8a). The expression profiles of VECs and LECs were compared between the NCWs and CWs and cellular processes in which these DEGs are overexpressed were analyzed (Figure 8b–e, Tables S7 and S8).

Enrichment analysis showed that several genes involved in the electron transport chain and ATP production, such as mt-Nd4 and mt-Cytb, were upregulated in both VECs and LECs in CWs. Whereas mitochondrial function is required for energy production, higher levels of mitochondrial function or disproportionate activity of the electron transport chain (ETC) can contribute to the high levels of OS observed in CWs. Both VECs and LECs showed significantly lower expression of heat shock protein family members, which can impair the response to the higher OS associated with CWs. Upregulation of Camk1d in both VECs and LECs in CWs can negatively impact cell migration and interferes with neovascularization [107]. In addition, VECs showed upregulation of Dusp1 in CWs, which halts cell cycle progression in endothelial cells, which in turn can interfere with angiogenesis [107].

Several genes that were differentially expressed in CWs can contribute to vessel formation; however, the role of these genes in vessel formation has been mostly studied in the context of tumor cells. Tumor cells have leaky and fragile microvessels [108,109]. This suggests different patterns of neovascularization between NCWs and CWs and that angiogenesis in CWs is more like in tumors. Combined with the lower number of endothelial cells in CWs, these data suggest that vasculature and blood circulation necessary for healing are impaired in CWs from the early stages after injury.

In addition to the lower number of endothelial cells and altered energy production in these cells in CWs, the crosstalk between the endothelial cells and other cell types can be negatively impacted in CWs [109]. For example, Fib 2 in CWs showed downregulation of Amot and upregulation of Gpr39 (Figure 5c). Angiomotin, the product of Amot, can promote endothelial cell migration and angiogenesis. However, Gpr39 can interfere with angiogenesis through suppression of endothelial cell proliferation, migration, and tube formation [110,111]. Upregulation of this gene in Fib 2 in CWs can contribute to disrupted angiogenesis in these wounds. Pathway analysis also showed other pro-angiogenic pathways that were disrupted in Fib2. Downregulation of Axin2, Mylk [112], Fbln1 [113], and Tmem204 [114] along with overexpression of Rgs4 interfere with angiogenesis and vascular endothelial growth factor (VEGF) signaling. Given the higher number of Fib 2 in CWs, these detrimental effects are more pronounced in CWs.

LECs showed lower numbers in CWs than NCWs (Figure 8a). These lower numbers of cells can result in less effective lymphatic drainage in CWs. Given the importance of lymphatic vasculature in the homeostasis of the wound microenvironment and suppression of inflammation through drainage of inflammatory cytokines [115], this observation underlines the importance of functional lymphatic drainage in wound healing, a lack of which may contribute to non-healing.

### 3.5. Immune Cell Composition Is Different Between NCWs and CWs

The role of the immune cells in wound healing has been established by numerous studies [116]. Several cytokines released by the injured cells along with damage-associated molecules (DAMPs) such as ROS, hydrogen peroxide, and lipid mediators [5] recruit immune cells, first neutrophils and then monocytes, from circulation. Neutrophils primarily help with eliminating the pathogens in the wound microenvironment by releasing antimicrobial agents from their granules and by phagocytosing pathogens [117,118]. Monocytes differentiate into proinflammatory macrophages (M1) in the wound tissue early after injury [119]. Immune cells also promote the proliferation of fibroblasts and keratinocytes by secreting growth factors [118].

**Figure 9:**
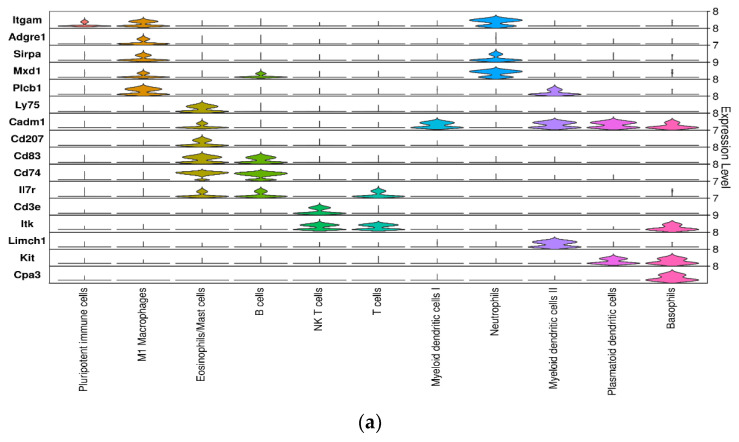

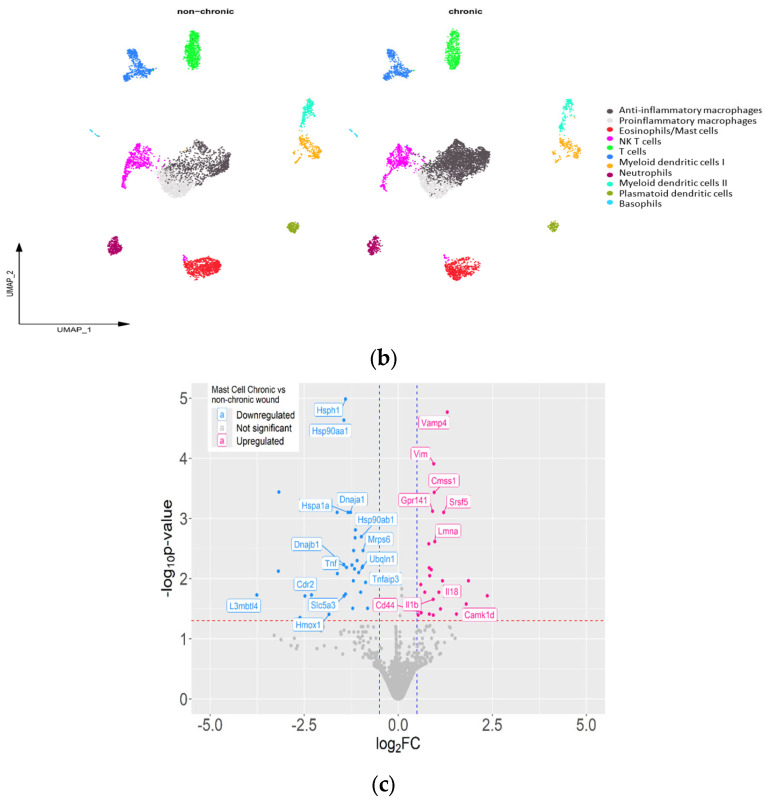
Anti-inflammatory macrophages are more abundant in CWs in early phases of healing. (**a**) Stacked violin plots showing cellular expression of immune cell markers in different clusters. (**b**) Comparison of the immune subclusters between NCWs and CWs. All immune cell types were more abundant in NCWs, with the exception of M2 macrophages, which are more abundant in CWs, highlighting the importance of immune cells and their composition in response to injury and healing. (**c**) Volcano plot showing genes differentially expressed in mast cells between NCWs and CWs. FDR < 0.05 and Log2FC > 0.5 in absolute value. Pink dots show genes upregulated in CWs and blue dots genes that are downregulated.

CWs have a prolonged inflammatory phase [59], but the specifics of the immune cell responses during the initiation of chronicity are not well understood. We identified several clusters in our original clustering to be immune cells and these clusters showed differences in cell numbers between NCWs and CWs (Figure 4). NCWs showed a higher number of M1 macrophages whereas CWs showed a higher number of M2 macrophages, which are anti-inflammatory. M1 macrophages are present in the early stages of inflammation in normal wound healing and secrete proinflammatory cytokines that recruit more immune cells to the wound microenvironment. In normal healing, M2 macrophages are present toward the end of the inflammatory phase and promote healing, scavenging cellular debris and promoting efferocytosis of the neutrophils that have eliminated the pathogens [118]. While both M1 and M2 macrophages were identified in the original cell clusters, several immune cells important for wound healing, such as neutrophils and mast cells, could not be identified among those clusters. Therefore, to obtain a more detailed understanding of the composition of various immune cells in NCWs and CWs, cells identified as immune cells in the original UMAP clustering were subclustered and classified using established immune cell-specific markers (Figure 9a,b). Similar to the original clustering of all cells, M1 macrophages were more abundant in NCWs, whereas M2 macrophages were more abundant in CWs (Figure 4 and Figure 9b). These findings parallel those by others who performed scRNAseq on human DFU tissue [65]. These investigators found that M1 macrophages were more abundant in healing ulcers, whereas M2 macrophages were more abundant among non-healing ulcers [65].

Among other immune subclusters, several subtypes, such as neutrophils and mast cells, were more abundant in NCWs. A comparison of the transcriptomic profile of these subclusters showed several genes to be differentially expressed with Log2FC > 0.5 in absolute value and *p*-value < 0.05 (Figure 9c, Table S9). Several of these genes contribute to the prolonged and higher levels of inflammation observed in CWs. Neutrophils eliminate DAMPs and pathogens through the release of the content of their vesicles [5]. Despite higher expression of neutrophil chemoattractants by several clusters in CWs, the neutrophils number was higher in NCWs. This discrepancy can be attributed to higher levels of OS in CWs that can lead to higher levels of apoptosis in neutrophils. In addition, CWs have higher numbers of M2 macrophages, which have higher efficacy in the efferocytosis of neutrophils. In addition to differences in the numbers of neutrophils between the two wound conditions, a comparison of the transcriptomic profile of these cells between NCWs and CWs showed several genes to be differentially expressed with Log2FC > 0.5 in absolute value and *p*-value < 0.05. Several of these genes contribute to the prolonged and higher levels of inflammation observed in CWs. For example, neutrophils showed upregulation of Sytl3 (Log2FC 8.66) in CWs which is involved in release of secretory vesicles. Whereas these processes are integral to the function of neutrophils in the elimination of pathogens and the release of growth factors, their increased levels in CWs can prolong inflammation by increasing the secretion of the inflammatory mediators and increasing OS.

Mast cells are another cell type found to be less abundant in CWs. In normal healing, mast cells help with clot formation during the homeostasis phase of wound healing by increasing the deposition of fibrin [120,121,122]. They also help eliminate pathogens by releasing enzymes such as granzyme B [123] and secreting inflammatory mediators that further recruit immune cells to the site of injury [121,122]. The function of these cells in wound healing, particularly in the early stages, heavily relies on their degranulation of mediators [5]. Differential expression analysis in mast cells with FDR < 0.05 and Log2FC > 0.5 in absolute value showed several DEGs between NCWs and CWs (Figure 9c, Table S9). In CWs, mast cells showed upregulation of Vim. Vimentin deficiency was shown to increase degranulation in mast cells [124]. Therefore, upregulation of vimentin in mast cells in CWs can negatively affect degranulation in these cells. Several heat shock proteins were downregulated in mast cells in CWs, including Hsp90aa1, Hsp90ab1, Hspd1, Hsph1, and Hspa1a [68]. Downregulation of these genes can lead to apoptosis of mast cells in CWs, which may explain their lower numbers in these wounds [125]. A lower number of mast cells coupled with their impaired degranulation will decrease the pro-healing effects of these cells in CWs. In addition, upregulation of several proinflammatory genes in mast cells, such as Il1b and Il18, can contribute to the high levels of inflammation observed in CWs.

Whereas a higher number of immune cells is conventionally associated with higher levels of inflammation, the composition of these cells as the wound becomes chronic suggests otherwise. During the initiation of chronicity, CWs have lower numbers of most immune cell types except for M2 macrophages. Furthermore, M2 macrophages are mostly considered as the immune cells that conclude inflammation and promote transition from the inflammatory phase to the proliferative phase [5]. Higher numbers of these macrophages in the CWs in the early stages of healing suggest that premature activation of monocytes to an anti-inflammatory phenotype may contribute to chronicity. In addition, these macrophages are also more abundant in well-established human DFUs that do not heal [65], which indicates that the pathways to chronicity are set shortly after injury.

## 4. Summary and Conclusions

**Figure 10:**
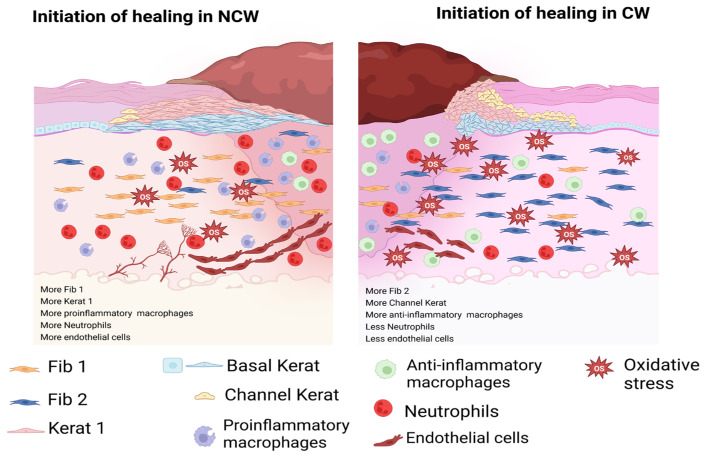
Summary of the differences in cell abundance between NCWs and CWs. CWs with higher levels of OS show higher number of Fib 2 (non-healing) and lower number of Fib1 (healing). Of the keratinocytes, the Basal Kerats and Kerat 1 (suprabasal keratinocytes) are much less abundant in CWs. An exception to this observation is the Channel Kerats, which are much more abundant in CWs. CWs showed lower number of neutrophils and M1 macrophages (proinflammatory) but had higher numbers of M2 macrophages (anti-inflammatory). Endothelial cells were more abundant in NCWs.

For these studies, we have used a mouse model of chronic wounds that mimics many features of chronic wounds in humans. These mouse wounds have high levels of OS, lack of re-epithelialization, chronic inflammation, impaired dermal–epidermal connectivity, damaged microvasculature (e.g., fibrin cuffs), abnormal collagen matrix in the wound tissue, biofilms that develop naturally, that is, without the introduction of exogenous bacteria, and do not heal without intervention [11,13]. Several studies have confirmed the presence of these features in human DFUs; however, all these studies find these features by comparing healing wounds with those in which chronicity is already established. Recently, one such study advanced our knowledge of the differences between chronic and healing wounds in diabetic patients to a single-cell level [65]. This study defines the role of different cell types and their function when CWs are fully chronic. The key to the understanding of chronicity resides in identifying how the different cells and genes important in healing are altered when chronicity ensues. It is this gap in the knowledge that our study fills. We found that during the initiation of wound healing, compared to CWs, NCWs have more pro-healing fibroblasts (Fib1) and keratinocytes (Kerat1), more M1 macrophages, more neutrophils, more mast cells, and more endothelial and lymphatic cells. CWs, on the other hand, have more non-healing fibroblasts (Fib2), more Channel keratinocytes, more M2 macrophages, fewer neutrophils and mast cells, and fewer endothelial and lymphatic cells (Figure 10).

**Figure 11:**
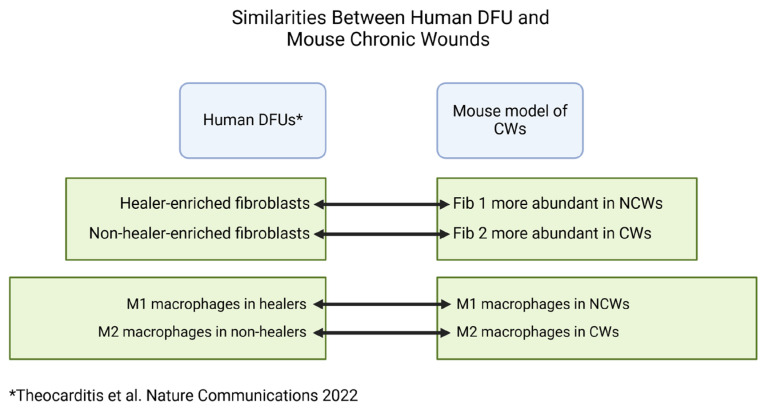
Mouse model of CWs shares important similarities with human DFUs. Human DFUs that ultimately heal show enrichment of “healer” fibroblasts whereas “non-healer” fibroblasts are enriched in wounds that do not heal [65]. A similar observation with Fib 1 and Fib 2 is present in our mouse model of CWs. In addition, in human DFUs that heal, M1 macrophages are more abundant, whereas M2 macrophages are more abundant in non-healing wounds. In our model, we also observe an enrichment of M2 macrophages in CWs, whereas M1 macrophages are more abundant in NCWs that heal.

The presence of high levels of OS in CWs, in conjunction with the findings presented here, have major implications in determining how to successfully treat chronic wounds because they not only reveal differences between the initiation of normal wound healing and the initiation of non-healing in chronic wounds, but they also reproduce findings from the scRNAseq study of well-established CWs in diabetic patients (Figure 11). Both studies show how cellular composition and major healing pathways contribute to prolonged inflammation and improper granulation tissue formation. These similarities in findings can be attributed to the several features of this mouse model that are also found in human DFUs. One such feature that can affect cellular composition of the CWs is the presence of bacterial biofilm, which unlike other models of impaired wound healing forms within the early stages of response to injury. Bacterial biofilms have been shown to polarize macrophages toward an anti-inflammatory phenotype through cyclic-di-AMP and STING (stimulator of interferon genes) interactions [126,127], which is reflected in our findings and those of human DFUs. In addition, similar to high levels of OS, bacterial biofilm can impede host fibroblast proliferation [128]. The findings from scRNAseq on human DFUs and our mouse model also suggest that these factors can affect fibroblast phenotype as well. We can now use this chronic wound model to determine how wounds become chronic by performing mechanistic studies in vivo coupled with bioinformatic studies to determine how cells interact with each other to affect the physiology of wound healing.

These studies, combined with previous studies we have performed on the development of biofilm in these mouse wounds, are also relevant to human chronic wounds because what we learn about the cellular composition and processes and the development of biofilm at the early stages of healing can be used in clinical settings by physicians to determine treatment of debrided ulcers.

Our findings provide insight into processes involved in the initiation of wound chronicity. These processes are complex and numerous, indicating that one type of treatment will not fix all the problems, unless the root cause is so fundamental to cell and molecular mechanisms, that correction of such a process would put a chronic wound on the path to healing. We suggest that such a fundamental process is high OS and its association with wound infection/biofilm.

## Figures and Tables

**Table 1:** Presence of hallmark cell markers in each of the clusters.

	*Ccr7*	*Csf3r*	*Cd86*	*CD247*	*Cdh3*	*Col1α1*	*Col3α1*	*Dcn*	*F13a1*	*Fit1*	*Gjb2*	*IL1b*	*IL1r2*	*Itk*	*Krt1*	*Krt14*	*LiLrb4b*	*MiTf*	*Pecam1*	*Prox1*	*Rgs1*	*S100a9*	*Scl7a11*	*Sema3a*	*Vim*
Basal Kerats																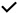									
Kerats 1																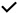									
Kerats 2					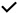											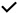									
Channel-related Kerats											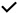					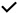									
Differentiated Kerats															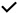										
Kerats 3																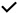									
Fibroblast 1						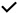		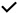										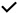							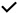
Fibroblast 2							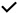	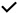										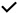							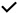
Macrophage 2			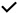						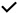								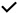	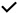							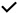
Macrophage 1			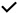									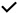	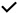												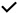
T cells				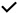										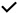							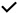				
LEC																				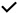				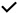	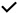
VEC										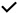															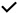
Melanocytes																		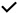							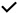
Immune cells	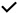	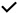	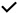						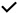			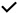	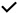										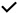		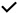
Unknown Immune cell 1		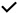																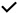					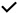		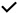
Unknown immune cell 2		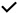										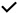	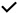				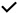					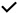	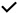		

Kerats—keratinocytes, LEC—lymphatic endothelial cells, VEC—vascular endothelial cells. 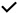 indicates the expression of the marker gene.

**Table 2:** Number of cells in each cluster in NCWs and CWs.

Cell Type (Cluster)	NCWs	CW	% Difference CWs vs. NCWs
Fibroblast 1	5918	4358	~26.4% fewer
**Fibroblast 2**	**1609**	**2553**	**~37.0% more**
Melanocytes	1105	823	~25.5% fewer
Keratinocyte 1	8936	5414	~39.4% fewer
Keratinocyte 2	4638	3366	~27.4% fewer
Keratinocyte 3	2975	2439	~18.0% fewer
Differentiating keratinocytes	3738	3197	~14.5% fewer
**Channel-related keratinocytes**	**1142**	**2728**	**~58.1% more**
Basal keratinocytes	1029	93	~91.0% fewer
Vascular endothelial cells	3470	3100	~10.6% fewer
Lymphatic endothelial cells	452	301	~33.4% fewer
Monocyte/macrophage 1	835	563	~33.6% fewer
**Monocyte/macrophage 2**	**2121**	**3441**	**~38.4% more**
T cells	1346	841	~37.5% fewer
**Immune cells**	**582**	**593**	**~1.8% more**
Unknown immune cells 1	791	477	~39.7% fewer
Unknown immune cells 2	309	228	~26.2% fewer

## Data Availability

Data are available upon request.

## Supplementary Materials

The following supporting information can be downloaded at: www.mdpi.com/article/10.3390/antiox14020214/s1, Table S1: Fib 2 vs. Fib 1 DEGs in NCWs. Table S2: Fib 2 vs. Fib 1 DEGs in CWs. Table S3: Fib 1 DEGs in CWs vs. NCWs. Table S4: Fib 2 DEGs in CWs vs. NCWs. Table S5: Kerat 1 DEGs in CWs vs. NCWs. Table S6: Channel Kerat DEGs in CWs vs. NCWs. Table S7: VECs DEGs in CWs vs. NCWs. Table S8: LECs DEGs in CWs vs. NCWs. Table S9: Mast cells DEGs in CWs vs. NCWs.
