# Supplementary material for: Chronic Wound Initiation: Single-Cell RNAseq of Cutaneous Wound Tissue and Contributions of Oxidative Stress to Initiation of Chronicity
Source: Antioxidants (Basel). 2025 Feb 13;14(2):214. doi: 10.3390/antiox14020214 (PMC11852160; doi:10.3390/antiox14020214)
Supplement: Supplementary file 1 [file antioxidants-14-00214-s001.zip › antioxidants-3353765-supplementary.pdf]

Supplementary Table S1. Fib 2 vs Fib 1 DEGs in NCWs.

Gene	Log_2FC	Gene	Log_2FC
Wnt2	3.9	Tshz2	3.8
Wnt5a	-3.8	Il1rl1	4.8
Wnt5b	-2.3	Il6	2.7
Sfrp5	4.8	Cxcl13	5.9
Pik3r1	-1.2	Cxcl14	4.5
Pik3r6	-3.9	Cxcl16	1.8
Pik3cb	-1.4	Cxcl1	4.4
Ctnna2	2.2	Csf2	5.6
Cdh2	4.4	Csf3	6.5
Cdh5	1.5	Ptgs2	2.2
Pcdh7	1.4	Itga2	2.8
Pcdh9	2.2	Plau	3.5
Pcdh19	2.8	Il33	3
Lamc3	-6.7	Lepr	-4.1
Rnd1	4.2	Gpr39	7.1
Megf6	-4.7	Axin2	-4
Smad6	-1.9	Mylk	-3.1
Bmp8a	3.7	Fbln1	-2
Bmp5	2.9	Tmem204	-3
Bmp4	-2	Adcy1	-3.6
Bmper	4.8	Adcy5	-2.3
Mmp11	-2	Adcy7	-1.3
Mmp16	-3.8	Adcyap1r1	-1.2
Timp1	1.4	Csmd1	5.2
Sulf1	3.7	Ptprj	3.2

Supplementary Table S2. Fib 2 vs Fib 1 DEGs in CWs.

Gene	Log_2FC	Gene	Log_2FC
Wnt2	5.8	Ptprj	3.5
Wnt5a	-2.7	Tshz2	3.8
Wnt5b	-3.2	Stat3	-0.9
Sfrp4	5.5	Jun	-1.5
Sfrp5	0.1	Il1rl1	4.0
Pik3r1	-1.7	Il6	2.2
Pik3r6	-1.4	Cxcl1	4.6
Pik3cb	-0.6	Cxcl2	5.3
Ctnna2	5.3	Cxcl3	7.2
Cdh2	5.9	Cxcl5	4.7
Cdh5	2.1	Cxcl13	3.5
Pcdh7	1.6	Cxcl14	4.0
Pcdh9	1.8	Csf2	4.4
Pcdh1	0.5	Csf3	4.4
Lamc3	-4.7	Ptgs2	2.4
Rnd1	4.8	Itga2	2.9
Megf6	-3.8	Plau	3.7
Smad6	-2.5	Rgs4	4.2
Bmp8a	4.7	Lepr	-2.7
Bmp5	3.7	Gpr39	6.8
Bmp4	-3.1	Axin2	-4.6
Bmper	5.0	Mylk	-3.3
Mmp11	-2.4	Fbln1	-1.8
Mmp16	-3.4	Tmem204	-2
Timp1	2.9	Adcyap1r1	-1.7
Adcy1	-5.4	Csmd1	4.8
Adcy5	-2.2	Sulf1	4.6
Adcy7	-1.6		

Supplementary Table S3. Fib 1 DEGs in CWs vs NCWs.

Gene	Log_2FC	Gene	Log_2FC
Hmox1	-1.6	Scube3	6.8
Hspb1	-1.1	Alpl	-2.1
L3mbtl1	-7.1		

Supplementary Table S4. Fib 2 DEGs in CWs vs NCWs.

Gene	Log_2FC	Gene	Log_2FC
Mmp14	1.11	Adamts14	1.17
Camk1d	2.3	Inhba	1.6
Spock2	1	Tafa2	2.6
Ank3	1.4	Rps20	1.3
Tpm2	1.8	Ddit4	1.9
Sdc1	1.3	Sdc1	1.3
Mrps6	-1.22	Cox7a2l	2.15

Supplementary Table S5. Kerat 1 DEGs in CWs vs NCWs.

Gene	Log_2FC	Gene	Log_2FC
mt-Nd1	1	Hspa4l	-1
mt-Nd4	0.6	Hsph1	-2.5
mt-Nd5	0.6	Hspb8	-1
mt-Co1	0.6	Dusp6	1.4
Fas	-1.3	Gjb4	1.8
Camk1d	2.4	Krt16	1.3
Cxcl14	1.3		

Supplementary Table S6. Channel Kerat DEGs in CWs vs NCWs.

Gene	Log_2FC	Gene	Log_2FC
Igfbp3	-1.1	Ets1	-1
Ptpn14	-1.4	Lgals7	0.9

Supplementary Table S7. VECs DEGs in CWs vs NCWs.

Gene	Log_2FC	Gene	Log_2FC
mt-Nd4	0.7	mt-Cytb	0.7
Camk1d	-1.5	Hspa1b	-0.8
Hspd1	-0.6	Hsp90aa1	-0.6

Supplementary Table S8. LECs DEGs in CWs vs NCWs.

Gene	Log_2FC	Gene	Log_2FC
Hspa1b	-0.8	Hsp90aa1	-0.6
Hspd1	-0.6	mt-Nd4	0.7
mt-Cytb	0.7		

Supplementary Table S9. Mast cells DEGs in CWs vs NCWs.

Gene	Log_2FC	Gene	Log_2FC
Vim	0.9	Hsp90aa1	-0.9
Hspd1	-1.5	Hsph1	-1.4
Hsp90ab1	-0.9	Hspa1a	-1.33
IL18	1		
